# Supplementary material for: Stabilization of the Quadruplex-Forming G-Rich Sequences in the Rhinovirus Genome Inhibits Uncoating—Role of Na+ and K+
Source: Viruses. 2023 Apr 19;15(4):1003. doi: 10.3390/v15041003 (PMC10141139; doi:10.3390/v15041003)
Supplement: Supplementary file 1 [file viruses-15-01003-s001.zip › viruses-2327039-supplementary.pdf]

Supplementary Figure S1

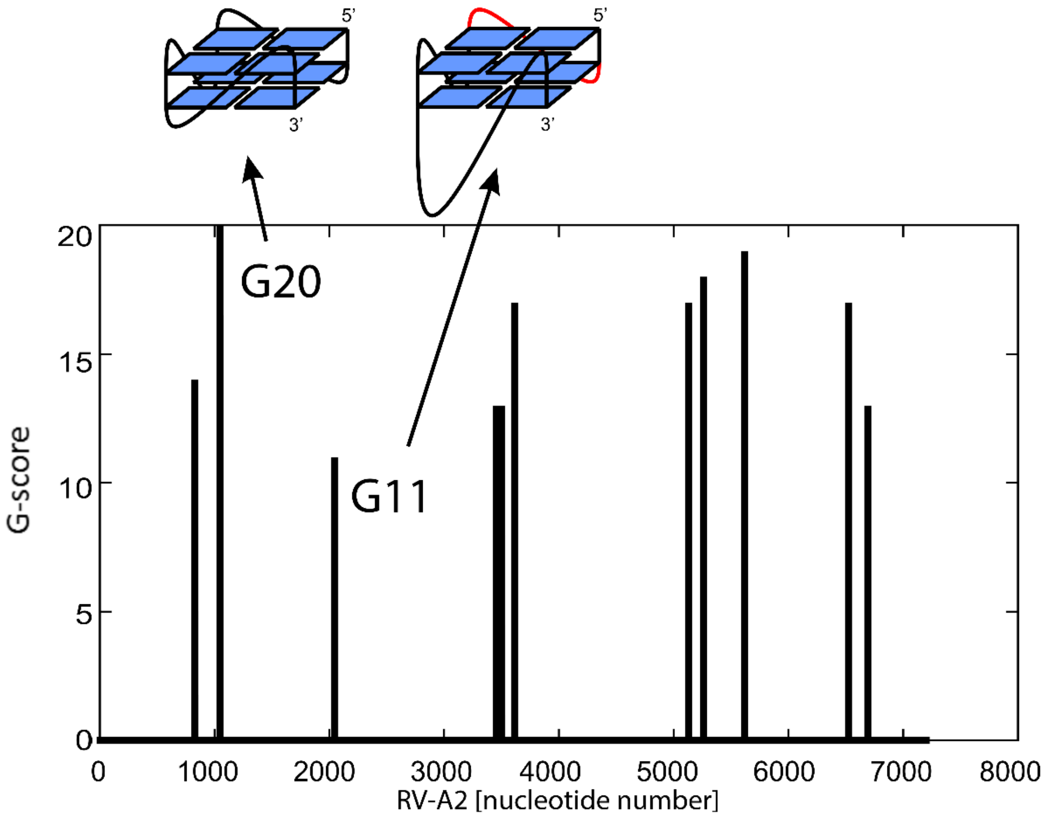

Supplementary Figure S1. QGRS-mapper analysis of RV-A2 RNA. Regions with G-scores $\geq$ 10 within non-overlapping windows of 30 nucleotides. Positions of the oligonucleotides G11 and G20 derived from the RV-A2 sequence are indicated. Top, cartoons of putative folds of G11 and G20 GQs. Note the long loop and putative zero-loops (red) in G11.

Supplementary Figure S2

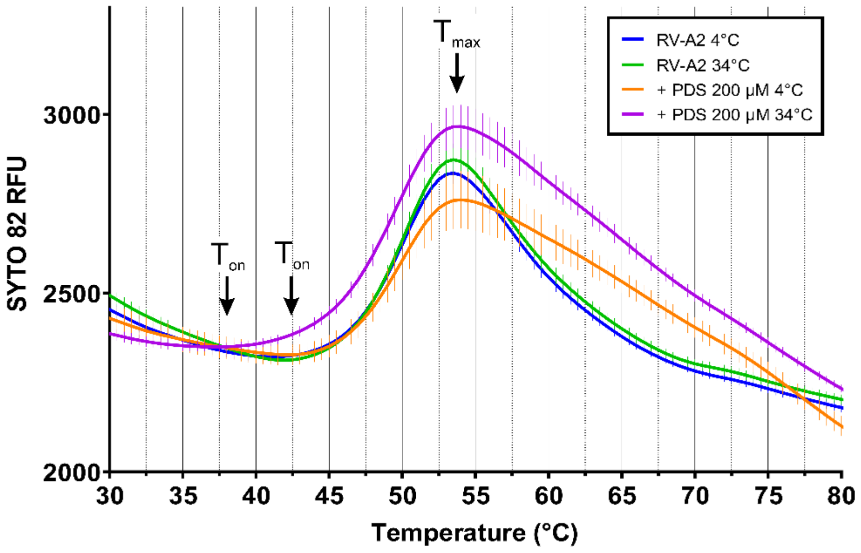

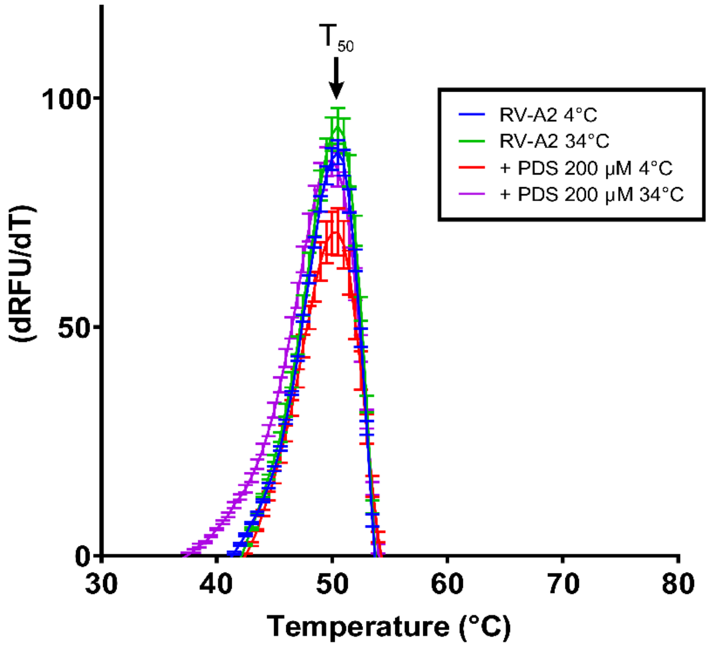

Supplementary Figure S2. PaSTRy analysis of purified RV-A2 preincubated with 200 µM PDS. For the mean values of temperature-dependent RNA accessibility see Fig. 3A. Purified virus was heated in a real-time PCR machine from 25 to 95 °C at 1.5 °C/min ramp rate in the presence of Syto 82. Samples were excited at 541 nm and the emission intensity was measured at 560 nm every other 0.5 °C temperature increase. The relative fluorescence units are plotted against the temperature.

Supplementary Figure S3

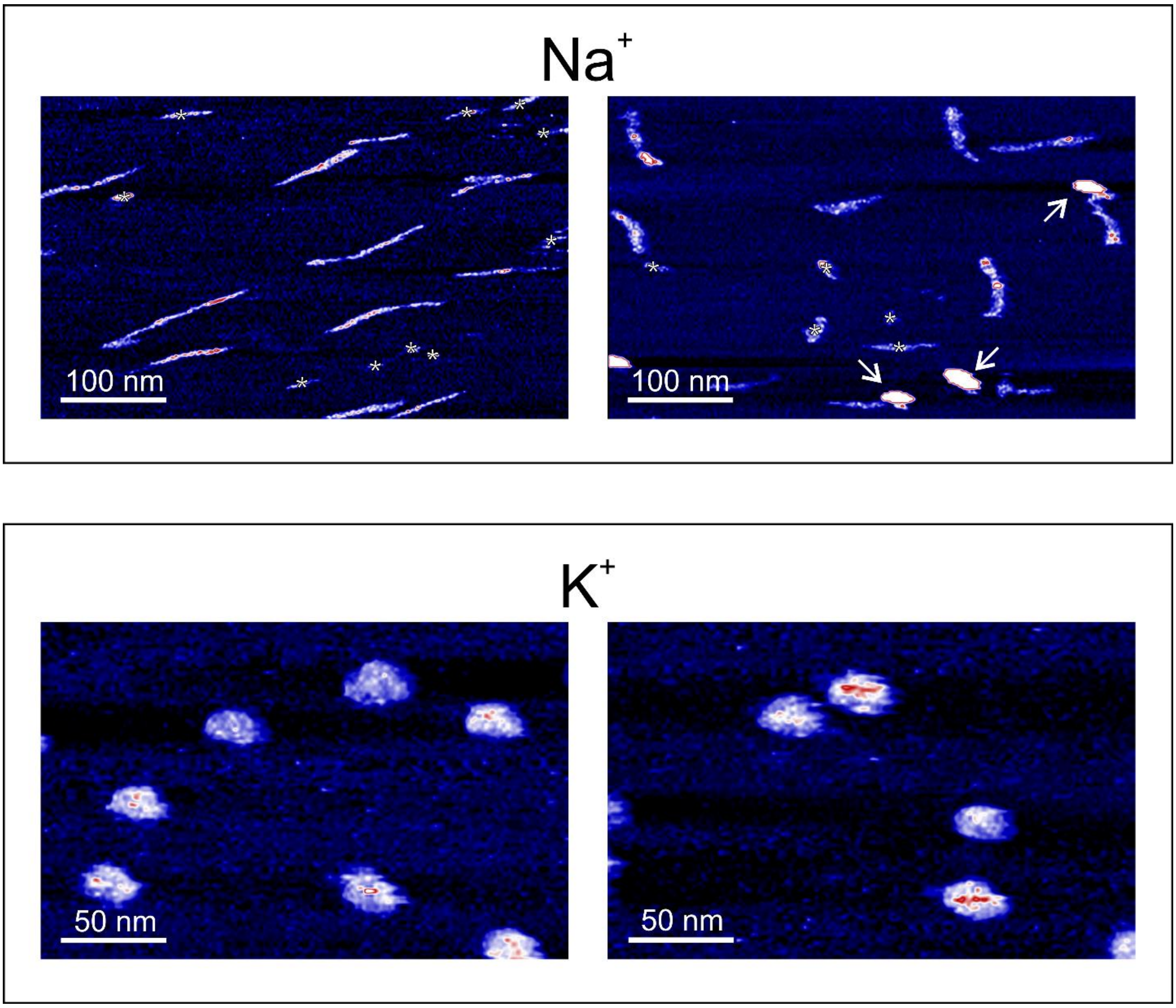

Supplementary Figure S3. AFM imaging of RNA cores in the presence of PDS in Na^+ or K^+ phosphate buffer. RV-A2 RNA cores were incubated with PDS at a final concentration of 20 µM in 100 mM Na^+ or K^+ phosphate buffer as indicated for 10 min at 25 °C, applied to freshly cleaved mica and imaged. Asterisks indicate partially unwound RNA. Arrows point to RNA aggregates.

Supplementary Figure S4

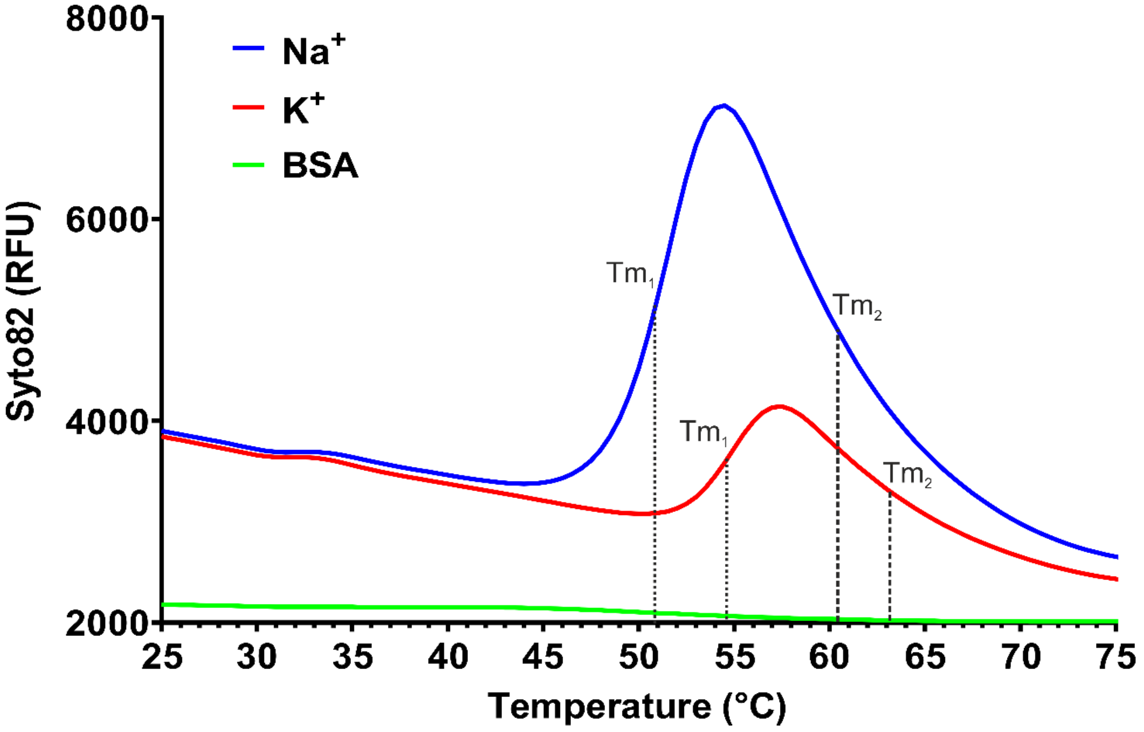

Supplementary Figure S4. Analysis of the stability of RV-A2 RNA cores in Na^+ or K^+ phosphate buffers. RNA cores left after mild proteolysis of RV-A2 capsids with proteinase K were heated in 100 mM Na^+ or K^+ phosphate buffer containing 5 µM Syto 82 in a real-time PCR machine from 25 to 95 °C at a 1.5° C/min ramp rate. Samples were excited at 541 nm, and the emission intensity was measured at 560 nm at each 0.5 °C temperature increase. Relative fluorescence units are plotted against the temperature. A sample containing BSA instead of RNA cores and treated similarly was used as a control to detect any possible interference of contaminant proteins with the Syto 82 signal.
